# The Effects of Lactose, Microcrystalline Cellulose and Dicalcium Phosphate on Swelling and Erosion of Compressed HPMC Matrix Tablets: Texture Analyzer

**Published:** 2010

**Authors:** Bendgude Namdeo Tukaram, Iyer Vidaya Rajagopalan, Poddar Sushi Ikumar Shartchandra

**Affiliations:** a *Department of Pharmaceutics, Principal K. M. Kundnani College of Pharmacy, 23 Jote Joy Bldg. Cuffe Parade, Colaba, Mumbai-400005. India.*

**Keywords:** Diltiazem HCl, Texture analysis, Swelling, Erosion, Hydroxy propyl methyl cellulose (HPMC), Solute transport

## Abstract

This paper reviews the use of texture analysis in studying the performance of hydrophilic matrices of highly soluble drugs and different types of excipients (*i.e. *water-soluble, water-insoluble and swellable, and water insoluble and non-swellable). Tablets were prepared by direct compression, and their swelling and erosion in presence of these different excipients were assessed with the help of volumetric, gravimetric, morphological, and rheological studies. Dissolution test was performed using USP 26 apparatus 2 modified by insertion of a sieve to prevent sticking of the tablets to the bottom of the vessel and allow them to swell 3-dimensionally. Loading 15% of the highly soluble drug in formulations containing 65% lactose showed the most pronounced swelling and erosion and the best sustained drug release, compared to matrices containing microcrystalline cellulose and dicalcium phosphate. The correlation between front movement, mass erosion and solute transport in relation to excipient type on progression of probe displacement and total work was examined throughout texture analysis studies. The formulation containing the soluble excipient lactose showed better swelling and erosion properties compared to formulations containing the swellable and insoluble excipients. In conclusion, it could be said that based on the distinct conventional dosage forms insertion of particular excipients in hydrophilic controlled release tablets containing water soluble drug, the finger print information of drug release profile could be obtained. To study the release profile from hydroxy propyl methyl cellulose K 15M matrices with different types of excpients, diltiazem hydrochloride was used as a model soluble drug.

## Introduction

The oral route of drug administration is typically considered the most preferred and convenient by patients. Modified-release technologies offer an effective means to optimize the bioavailability and the blood concentration-time profiles of various drugs. Monolithic matrices continue to be extensively used for preparation of oral modified-release delivery systems owing to their simplicity and ease of manufacture by using conventional processing equipments. Matrix systems generally consist of dissolved or dispersed drug within a swelling or slowly eroding polymer matrix. The drug release from such systems is controlled by water penetration into the matrix followed by either diffusion of the drug into the surrounding medium, erosion of the matrix, or combination of both. A potential disadvantage of the simple monolithic matrix system is the lack of zero-order release kinetics resulting from time-dependent changes in the diffusion path length and surface area. Such delivery systems are widely used to control the drug release due to their low cost, broad FDA acceptance, ease of manufacturing, favorable *in-vivo *performance and wide range of physicochemical properties which help modulate the drug release kinetics (1, 2). Hydrophilic polymers are widely used in controlled release systems due to their favorable functionality. At the molecular level, the drug release rate from polymer matrix is determined by the polymer swelling front, drug dissolution diffusion, and matrix erosion. These occur by the interaction of water molecules with polymer matrix and drug molecules. Water should penetrate into the polymer chains which leads to polymer swelling and drug dissolution before the drug can diffuse out from the matrices (3, 4). This results in a decrease in glass transition temperature of the polymer to the experimental temperatures (HPMC Tg of 84 °C to lower than 37 °C). This indicates transformation of the glassy state of the polymer into a rubbery gel (5). According to the explained mechanism, enhancing the mobility of the polymer chains and diffusing of the drug out from such polymer matrices could be done by inclusion of different types of excipients at different concentrations. 

The purpose of this study was to investigate the influence of the excipient type on matrix hydration, erosion and drug release from hydroxy propyl methyl cellulose K 15M based matrix systems with/without a highly soluble drug and different concentrations of water-soluble and swellable/non-swellable water-insoluble excipients. These studies were done with the help of volumetric, morphological and gravimetric methods (6). The whole data was correlated with the results from texture analysis studies of selected concentrations of these excipients (65% lactose, microcrystalline cellulose and dicalcium phosphate). The effects of the water-soluble, swellable and non-swellable excipients on transport of the solute, gel strength and boundary movements were also discussed in comparison with the control tablets containing no excipient.

## Experimental

*Materials*

Diltiazem HCl was obtained from Themis laboratory, Mumbai, India. Hydroxy propyl methyl cellulose K 15M, was procured from Colorcon Asia Pvt. Ltd. Goa, India. Lactose monohydrate, microcrystalline cellulose (Avicel PH 101) and anhydrous dicalcium phosphate was obtained from Evonics pharmaceutical, Mumbai, India. All other ingredients used throughout the study were of the analytical grade.

*Preparation of matrix tablets*

Ten different batches of 450 mg tablets containing hydroxy propyl methyl cellulose K 15M along with lactose, microcrystalline cellulose or dicalcium phosphate were prepared by using 13.5 mm punches for volumetric, morphological, gravimetric and rheological studies. Four different batches of diltiazem hydrochloride tablets were also prepared each containing 90 mg of the drug. The polymer: excipient ratio was developed on the basis of the above-mentioned studies to adjust the drug release profile and to keep the total weight of the matrix tablets constant at 450 mg for all batches under the experimental conditions of preparation (Table 1). The ingredients were passed through an 80 mesh sieve and mixed in a blender (kamavati, India). Tablets were compressed by direct compression (DC) with a rotary tablet press (jaguar, India) using 8.5 mm diameter circular flat punches at such a compression force that a predetermined hardness (5-6 kg/cm^2^) was achieved.

**Table 1:** Composition of ten different batches of lactose at ten different concentration levels

**Ingredients **	**Formulations (% compositions) **									
	F1	F2	F3	F4	F5	F6	F7	F8	F9	F10
**HPMC K 15M **	05	10	19.5	34.5	49.5	69.5	79.5	84.5	89.5	94.5
**Lactose **	93	88	78.5	63.5	48.5	28.5	18.5	13.5	8.5	3.5
**Magnesium stearate **	0.5	0.5	0.5	0.5	0.5	0.5	0.5	0.5	0.5	0.5
**Talc **	1.5	1.5	1.5	1.5	1.5	1.5	1.5	1.5	1.5	1.5

**Note: **Same batches of MCC and DCP were prepared on exact same manner

*Evaluation of tablets*

The prepared matrix tablets were evaluated for hardness, weight variation, thickness and friability. The hardness of the tablets was tested using Monsanto hardness tester. The friability values were determined using a Roche friabilator. The thickness was measured by Vernier calipers, and the weight variation test was performed according to IP method. 

*Swelling behavior of hydroxy propyl methyl cellulose K 15M (Volumetric Study) (* 7 *,* 8 *)*

The swelling diameters of the hydroxy propyl methyl cellulose K 15M tablets and the tablets containing the polymer with water-soluble or water-insoluble excipients were measured by keeping the tablets in dissolution medium (distilled water) (Table 1). Care must be taken to ensure that the tablets get sufficient dissolution medium to swell properly. Studies were done by dynamic method for six h. Tablets were hourly removed from the dissolution medium and the swelling path lengths of the gel layers were measured with the help of a ruler. 

*Swelling behavior of hydroxy propyl methyl cellulose K 15M (morphological study) (* 8 *,* 9 *)*

The swelling behaviors of the hydroxy propyl methyl cellulose K 15M tablets and the tablets containing the polymer with water-soluble or water-insoluble excipients were studied by dynamic method (dissolution apparatus) (Table 1). Initially tablets were stuck to the cover slip with the help of cyanoacrylic adhesives and then put into the dissolution jar. Tablets were hourly removed from the beaker/dissolution jar and image analysis was done with the help of the digital camera DC E300 keeping the background perfectly black. 

*Swelling behavior of hydroxy propyl methyl cellulose K 15M (rheological study) (* 10 *)*

The swelling behaviors of the hydroxy propyl methyl cellulose K 15M tablets and the tablets containing the polymer, diltiazem hydrochloride, and the water-soluble, swellable and water-insoluble excipients like lactose, microcrystalline cellulose and dicalcium phosphate were studied by dynamic method (dissolution apparatus USP 2) (Table 2). 

**Table 2:** Composition of Diltiazem HCl matrix tablets

**Ingredients **	**Formulations (% Compositions) **				
	A*	A	B	C	D
**Diltiazem HCl **	00	15	15	15	15
**HPMC K 15M **	98	18	18	18	83
**Lactose **	00	65	00	00	00
**MCC **	00	00	65	00	00
**DCP **	00	00	00	65	00
**Magnesium stearate **	0.5	0.5	0.5	0.5	0.5
**Talc **	1.5	1.5	1.5	1.5	1.5

**Note: **One more formulation prepared for texture analysis for comparison purpose contains 98% of HPMC K 15M, 0.5% magnesium stearate and 1.5% of talc (A*).

Initially tablets were stuck to the cover slip with the help of cyanoacrylic adhesives and then put into the dissolution jar.

Then, the hydrated tablets were removed at predetermined intervals, put on tissue papers and subjected to texture profiling to determine the gel layer thickness and study the movement of the swelling and erosion fronts and the total probe penetration into the entire swelled matrix up to the maximum penetration at constant force. Texture analysis was performed using TA.XT Plus texture analyzer equipped with 500 g load and texture expert software (10). The force-displacement-time profiles were obtained from penetration of the 2 mm diameter stainless steel probe into the swollen matrices at a data acquisition rate of 250 points per second. Probe approached the sample at a pretest speed of 2 mm/sec. Once a trigger force of 0.7 g was detected (upon contact of the probe and tablet), the speed of the advancing probe was changed to the test speed of 0.2 mm/sec until the maximum force was reached. The thickness of the swollen tablet could be determined by measuring the total probe displacement value recorded. The percent axial swelling could be calculated according to the following equation 1:

Axial swelling (%) = swollen thickness – original thickness/original thickness ×100          (1)

Swollen gel thicknesses measured by texture analyzer predict the entire free axial swelling thicknesses of the matrices without any constrain exerted on tablets. Texture analyzer is a novel approach to investigate the gel strength of the swollen tablets and compare the results with the swelling behavior of tablets observed in volumetric study (which is discussed earlier in this paper). The total work of penetration, which measures the gel strength in response to the resistance generated during probe displacement, was determined using equation 2:

The total work done through penetration = WT = ∫ F d D          (2)

W = Work done 

F = Force applied

d = Diameter of the probe

D = Distance traveled

*Water uptake and erosion studies (gravimetric evaluation)*

The uptake of water by tablets is a crucial step for *in-situ *gelling, *i.e*. transformation into gel and for subsequent swelling and erosion of the polymer matrix. Water uptake and erosion were studied on one h intervals up to six h. The following equations were used to determine the percent weight gain or water uptake, and the percent mass loss or erosion (11, 12):

Weight gain (%) = wet weight - dry weight/dry weight × 100          (3)

Weight loss (%) = original weight - dry weight/original weight ×100           (4)

*Release study*

The *in-vitro *dissolution studies were carried out using USP 26 dissolution apparatus type 2 (paddle method) at 50 rpm, 900 mL of distilled water as the dissolution medium, up to 8 h at 37 ± 0.5 °C. Five mL aliquots were withdrawn and replaced with the same volume of the medium at regular intervals. The withdrawn samples were filtered through 45 μ membrane filter (PVDF). After suitable dilution, the drug contents were determined spectrophotometrically at 238 nm by the above-mentioned method. The drug contents in samples were obtained using a standard calibration curve for diltiazem hydrochloride.

## Results and Discussion

*Volumetric evaluation*

**Figure 1:**
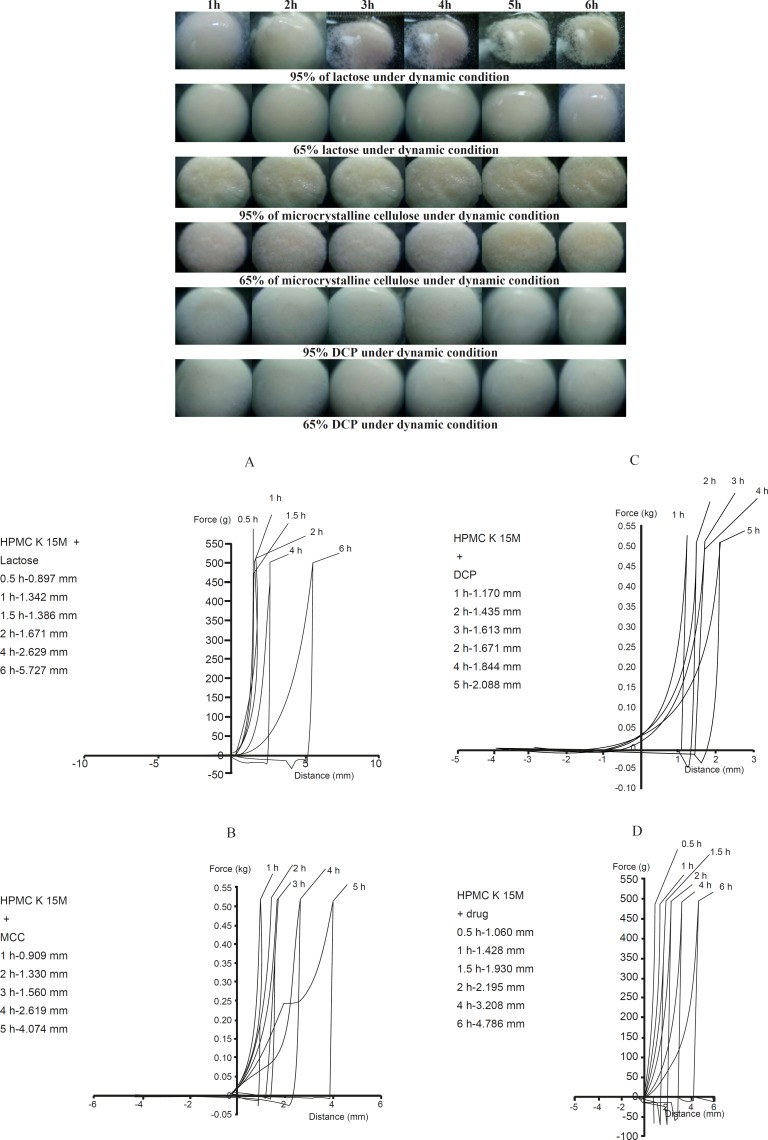
Morphological behavior of swelling and erosion of HPMC K 15M at different concentration level of Lactose, MCC and DCP (Dynamic Study).

Diameter changes of the hydroxy propyl methyl cellulose K 15M tablets were recorded with the help of a ruler. Ten formulations containing different concentrations of lactose, microcrystalline cellulose and dicalcium phosphate along with the polymer were taken for dynamic studies and the results were represented as a plot of diameter change against time. Swelling and erosion were observed only at higher concentrations of these excipients. There was no significant difference between the lactose, microcrystalline cellulose, and dicalcium phosphate-containing formulations at the initial stage of swelling. However, the subsequent rate of hydration in lactose-containing formulation was greater than that of the microcrystalline cellulose- and dicalcium phosphate-containing formulations due to water solubility of lactose. On the other hand, microcrystalline cellulose is a water-swellable polymer and swells for a long period of time. Therefore, it showed greater swelling diameter after 6 h as compared to lactose-containing formulations (Figure 1). 

Lactose dissolves and leaves behind pores to be filled with more water, so results in more penetration of water into the matrix. As the concentration of lactose went on increasing, an increase in water uptake by the polymer matrix and ultimately an increase in swelling were observed (13, 7). This was up to lactose concentrations of lower than 65% in polymer matrices; beyond that, increase in water uptake along with increase in erosion of the polymer matrix favored. This effect became more prominent if the concentration continued to increase: the formulation containing 95% of lactose got swelled and eroded within 5 h. On the other hand, the formulations containing microcrystalline cellulose and dicalcium phosphate showed steady erosion rate on increasing concentration, due to their lower water uptake capacity and being less porous. Dicalcium phosphate formed a more compact blend with hydroxy propyl methyl cellulose K 15M and showed less swelling and erosion compared to microcrystalline cellulose and lactose (Figure 1). These swelling and erosion behaviors of hydroxy propyl methyl cellulose K 15M in presence of lactose, microcrystalline cellulose and dicalcium phosphate were confirmed with the help of texture analysis and are discussed under “Rheological Study”. Studies indicated that the higher concentrations of polymer in formulations favored swelling while lower concentrations of the polymer (higher concentrations of the excipients) favored erosion (8, 9). In case of drug loaded formulations, significant increase in drug release rate was observed only when the concentrations of these excipients exceeded 65%.

*Morphological evaluation*

Morphological studies on swelling and erosion of lactose-, microcrystalline cellulose- and dicalcium phosphate-containing formulations were done with the help of DC E300 digital camera. For this purpose, ten different concentrations of lactose, microcrystalline cellulose and dicalcium phosphate in formulations were prepared for volumetric and gravimetric studies. Photographs of tablets containing 95% and 65% lactose, microcrystalline cellulose, or dicalcium phosphate freshly removed from the dissolution medium in an hourly basis, were taken. As lactose is a water-soluble excipient, forms more micro-cavities in polymer matrices, which helps explain the higher swelling and erosion (Figure 1) (14). Microcrystalline cellulose is a water-swellable polymer which swells with hydroxy propyl methyl cellulose K 15M, thus increasing the diffusion path length and reducing the drug release rate by time. 

Microcrystalline cellulose showed excellent compression properties and better plasticity as compared to lactose and dicalcium phosphate. On the contrary, dicalcium phosphate is a water-insoluble excipient which caused less prominent swelling, erosion, and drug release sustaining properties in matrices compared to lactose and microcrystalline cellulose. From this study, it is concluded that lactose-containing formulations (in concentrations of more than 65%) give higher release rates compared to formulations containing other excipients.

*Gravimetric evaluation:*

*Water uptake and mass loss study*

Generally polymer dissolution and erosion take place in three steps: Solvent penetration into the polymer matrix, polymer swelling and chain disentanglement and attainment of the threshold disentanglement. When water penetrates into the polymer matrix, it enhances the mobility of the polymer chains which eventually disentangle at the advancing front, separating the gel layer from the erosion/dissolution front. 

When the concentration of lactose increased, the enhanced osmotic pressure accelerated water penetration into the matrix resulting in a higher degree of polymer swelling and formation of more micro-cavities. Gravimetric studies on hydration and mass loss revealed that the rate and the extent of water uptake were significantly greater in lactose-containing compared to microcrystalline cellulose- and dicalcium phosphate-containing formulations. Water uptake study was carried out by calculating the percent weight gain and the percent remaining using formulas 3 and 4. Percent weight gains in lactose-containing hydroxy propyl methyl cellulose K 15M matrices were greater than the microcrystalline cellulose-containing and the dicalcium phosphate-containing formulations because of the greater water absorption capacity of hydroxy propyl methyl cellulose K 15M in presence of lactose. Lactose is water-soluble, so it produces more pores in contact with water forming more micro-cavities during the various mesophase formation, and causes the osmotic effect which promotes the swelling and erosion of hydroxy propyl methyl cellulose K 15M and drug release from the matrices (11, 12). 

Similar to the discussion in volumetric study, the initial higher concentration of the polymer absorbed water with the help of the matrix-embedded lactose and showed an increase in percent weight gain with time; however, the reverse is observed when lactose concentration increased, since the increase in water uptake caused polymer erosion and a decrease in percent weight gain. According to the gravimetric studies (Dynamic method) of lactose, microcrystalline cellulose and dicalcium phosphate, all three excipients were able to absorb water in contact with the dissolution medium, with the percent weight gain being in the order of lactose > MCC > DCP. In case of the percent remaining weights, the order changed to MCC > DCP > lactose, because of the more elastic and swellable nature of MCC which promoted a better water absorption over a long period of time compared to lactose and DCP, resulting in a slower erosion rate within the same period of time (15- 17).

*Rheological study*

*Texture analysis*

Rheological study of the swelling hydroxy propyl methyl cellulose K 15M formulations containing lactose, microcrystalline cellulose or dicalcium phosphate with the highly soluble drug diltiazem hydrochloride was performed by texture analysis of the swollen tablets and the results were compared with those of the volumetric, morphological and gravimetric studies. From the volumetric, gravimetric and morphological studies it was observed that hydroxy propyl methyl cellulose K 15M showed the best swelling and erosion properties only when higher percentages of the excipients were present. 

**Figure 2:**
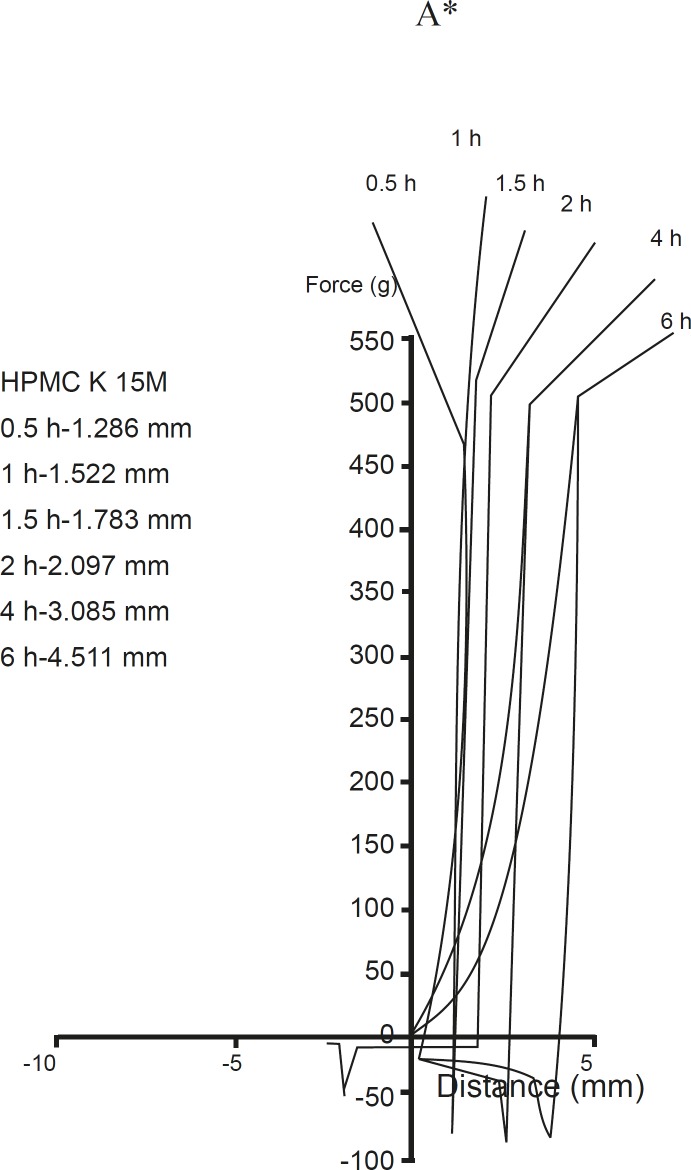
Force probe-displacement profiles for the formulation A, formulation B, formulation C, formulation D and formulation A* at different time interval. The initial resistance to probe penetration in to core (up to 1.5 h) and overall tablet thickness up to 5.727, 4.074, 2.088, 4.786 and 4.511 of formulation A, B, C, D & A* respectively

**Figure 3:**
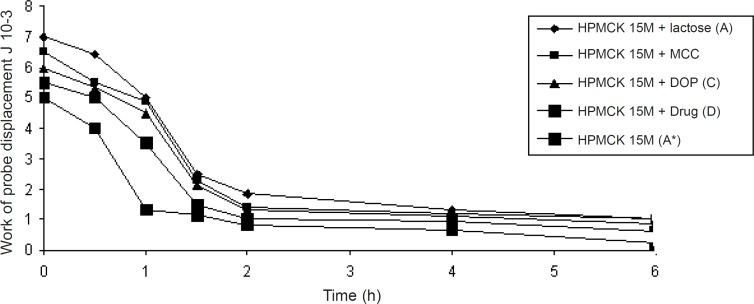
Total work of probe displacement at different time point for the formulation A, A*, B, C, and D.

Polymer swelling occurs as a result of the osmotic stress exerted at the advancing glassy core and rubbery gel. When the drug/excipient solubility increases, the enhanced osmotic stress causes more water penetration into the matrix resulting in a higher degree of polymer swelling. Figure 2 shows the texture analysis (TA) profiles for A, B, C, D and A* formulations obtained at different time intervals after the matrices were exposed to dissolution medium under the same conditions as in dissolution studies. The force required for the probe to penetrate into the swollen tablet decreased with time, as the swelling proceeded and the gel strength was reduced (Figure 3). The force increased once the probe crossed the boundary of the eroding part and started to get entry into the swelling glassy core (18, 19). This would help to better understand the solute transport in presence of lactose, microcrystalline cellulose and dicalcium phosphate in different formulations. The present illustration indicates that the initial drug release from such matrices occurred only by the diffusion mechanism followed by a release stage caused by both swelling and erosion. 

**Figure 4:**
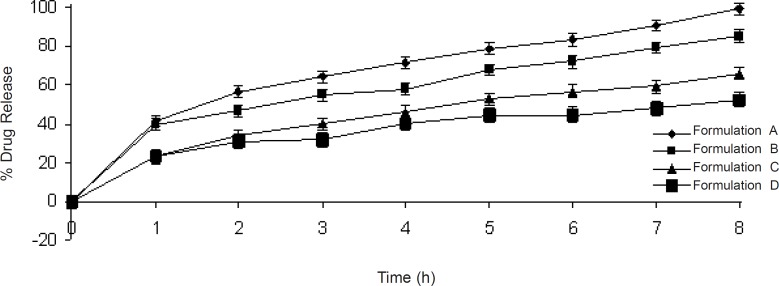
Diltiazem HCl Release profile from formulation containing lactose (A), MCC (B), DCP (C), and Plane polymer (D).

It is concluded that matrices showing higher swelling for a long period of time followed by erosion, are good candidates for preparation of controlled-release formulations of highly soluble drugs. Also, polymers which show better swelling as well as erosion properties are good candidates for low solubility drug. Figures illustrate that there is more steady water uptake from the formulations containing highly soluble drugs as compared to other formulations, while the highest swelling degree was observed in a lactose-containing formulation. This indicates that lactose showed the greatest water uptake capacity compared to the other excipients as well as the highly soluble drug. 

Obtaining the applied force and the probe displacement values, the total work required for probe penetration was calculated according to equation 2. During 1 h and 6 h, formulations A and D showed lower values of work of penetration, which is attributed to the soluble nature of both lactose and the drug so promoting greater water penetration and subsequently weakening of the gel structure as shown in Figures 2 (A, D) and 3. In case of formulations A*, B and C containing the polymer, microcrystalline cellulose, and dicalcium phosphate respectively, the gel strength values were greater over the same period of time. The inward movement of the fully hydrated region boundary as well as the increase in total thickness of the swollen tablets for each time point is apparent in all TA profiles as shown in Figure 2 (20). 

*Release study*

Diltiazem hydrochloride tablets showed good weight variation (SD values from 0.6 to 0.88) and content uniformity (98.73–103.60% w/w). Friability was 0.94% w/w, which was acceptable according to the USP 2006. As discussed earlier, the Korsmeyer-Peppas equation (M_t_/M_∞_ = Kt^n^) is the best equation to study the *in-vitro *drug release profile in such matrix formulations. The main parameters for Korsmeyer-Peppas equation (K and *n *values) were obtained from linear regression analysis performed by Microsoft Excel software to fit the dissolution data. For calculation purpose, the equation was modified as: 

log (% drug release) = log k + *n *log t (time)            (5) 

The *n *values indicate that which mechanism is prominent in drug release from the matrices. All formulations (*i.e*. A, B, C and D) followed the Quasi-fickian diffusion mechanism as the *n *values for these formulations were less than 0.5 (Table 3). In case of formulation A, the best fitted model was Korsmeyer-Peppas as it had a correlation coefficient (r) of 0.9973 which was greater than the value obtained from the zero-order, first-order, matrix and Hixson- Crowell models. For the same reason (*i.e. *having higher r values of 0.9661, 0.9989, and 0.9861 respectively) the best fitted model for formulations B, C, and D was matrix (Table 3). All these results were obtained from formulations at higher concentrations of the excipients (*i.e*. 65% of lactose, microcrystalline cellulose and dicalcium phosphate). These formulations were used to compare the release profiles to those of the formulation containing the polymer alone. Volumetric, morphological, gravimetric and rheological studies’results indicated that the lactose-containing formulation had the best hydration, swelling, and erosion properties, so the best release profile compared to formulations containing microcrystalline cellulose, dicalcium phosphate, and no excipient (containing only the polymer). 

**Table 3:** Mathematical modeling and drug release mechanism of Diltiazem HCl SR tablets

**Formulations **	**K **	***n ***	**R **	**% Release SD (n=3) **	**Model fitting **
**A **	41.92	0.3979	0.9973	99.31% (3.0)	Pappas
**B **	40.12	0.3305	0.9661	85.48% (3.0)	Matrix
**C **	23.59	0.4907	0.9989	66.07% (3.5)	Matrix
**D **	22.92	0.39	0.9861	52.78% (3.7)	Matrix

**Note: **Figures in parenthesis represents the ± SD

Dissolution results indicated that during 8 h, the lactose-containing formulation released more than 99% of the drug content, while the microcrystalline cellulose-containing, dicalcium phosphate-containing and no exicipient-containing formulations released 85.48, 66.07 and 52.78% respectively (Table 3). It is concluded that as lactose is a water-soluble excipient, it increases the hydration rate and relaxation of the polymer chains, resulting in more dissolved drug diffusing out from the matrix. Also when the drug solubility increases, the enhanced osmotic stress accelerates water penetration into the matrix resulting in a higher degree of polymer swelling and formation of more micro-cavities. Therefore, diltiazem hydrochloride in presence of lactose shows a proper release from the formulation. Microcrystalline cellulose is a swellable excipient and shows good swelling with hydroxy propyl methyl cellulose K 15M but less disentanglement with the polymer, so a declined solute transport compared to lactose. In case of dicalcium phosphate-containing formulation, there is no noticeable effect on swelling, erosion and hydration of hydroxy propyl methyl cellulose K 15M, and the release is prolonged as compared to lactose and microcrystalline cellulose-containing formulations. 

## Conclussion

Lactose, by its water-soluble and hydrophilic nature, facilitates gel formation and shortens the penetration time of the dissolution medium into the matrix. Moreover, this soluble substance acts as a channeling agent by rapidly dissolving and easily diffusing outward, therefore decreasing tortuosity and/or increasing the matrix porosity. Therefore lactose is considered as one of the most suitable excipients to achieve predetermined drug release profiles in controlled release matrices containing natural as well as synthetic polymers. The TA would provide additional operational tool to formulation scientists, allowing them to select optimal and easy to adapt methodology for dosage form development and assessment. 
